# Descending Colon Metastasis of Renal Cell Carcinoma: An Unusual Site of Metastasis

**DOI:** 10.7759/cureus.59756

**Published:** 2024-05-06

**Authors:** Ali Tariq Alvi, Elsa Lesley Tchouambou Pougoue, Cameron Summers-Powell, Mayuri Gupta

**Affiliations:** 1 Internal Medicine, HCA Florida Northwest Hospital, Margate, USA; 2 Pathology, HCA Florida Northwest Hospital, Margate, USA; 3 Gastroenterology, HCA Florida Northwest Hospital, Margate, USA

**Keywords:** metastasis to the colon, colonic mass, renal cell metastasis, clear cell cancer, renal neoplasm

## Abstract

Renal cell carcinoma (RCC) has a high metastatic potential. While metastasis to common sites like the lungs, liver, bones, and brain is well-documented, metastasis to the colon, particularly the descending colon, remains an uncommon occurrence. When RCC does metastasize to the gastrointestinal tract, it commonly spreads to the small bowel and stomach. There are few cases reported in literature involving RCC metastasis to the colon. The commonly affected areas within the colon include the rectosigmoid colon, splenic flexure, and transverse colon. We describe an 87-year-old male with a history of stage III RCC diagnosed three years ago, followed by left-sided nephroureterectomy, partial adrenalectomy, and perinephric lymph node dissection. He presented to the emergency department (ED) with melena and generalized abdominal pain for one week. Stool occult blood was positive. Computed tomography (CT) of the abdomen was significant for stable postsurgical changes related to prior left nephrectomy and colonic mass at the proximal descending colon. A colonoscopy revealed a necrotic appearing friable mass in the descending colon. The pathology of the mass revealed proliferated atypical cells positive for paired box 8 (PAX8), a cluster of differentiation 10 (CD10), RCC, and pan-cytokeratin and negative for caudal-type homeobox 2 (CDX2), thyroid transcription factor-1 (TTF-1), and a cluster of differentiation 68 (CD68), consistent with metastatic RCC.

## Introduction

Renal cell carcinoma (RCC) is a primary kidney tumor with a mortality of up to 40%, which is the highest among all genitourinary cancers [1]. Among the various histologic subtypes of RCC, clear cell cancer is the predominant one, accounting for 75-80% of all cases [2,3]. Metastatic disease is frequently present in patients with RCC, being diagnosed in almost 25% of patients [1]. When identified in its early stages, the cancer can be cured through surgical removal, although a small number of cases may still be susceptible to recurrence [2]. The metastasis has no cutoff time, with late metastasis observed in almost 10% of patients after five years. Likewise, nearly 40% of patients experience metastatic activity even after undergoing surgical removal [1,4].

The typical sites of metastatic spread in RCC include lymph nodes, liver, lungs, bone, brain, and adrenal glands [5,6]. Gastrointestinal tract metastasis is unusual, and only a few cases are reported in the literature in which RCC spreads to the colon following curative nephrectomy [1]. In our review of the existing literature, we did not encounter any cases of metastasis of RCC to the descending colon. Here, we describe a rare case of a patient with metastatic renal cancer to descending colon three years after surgical resection.

## Case presentation

**Figure 1:**
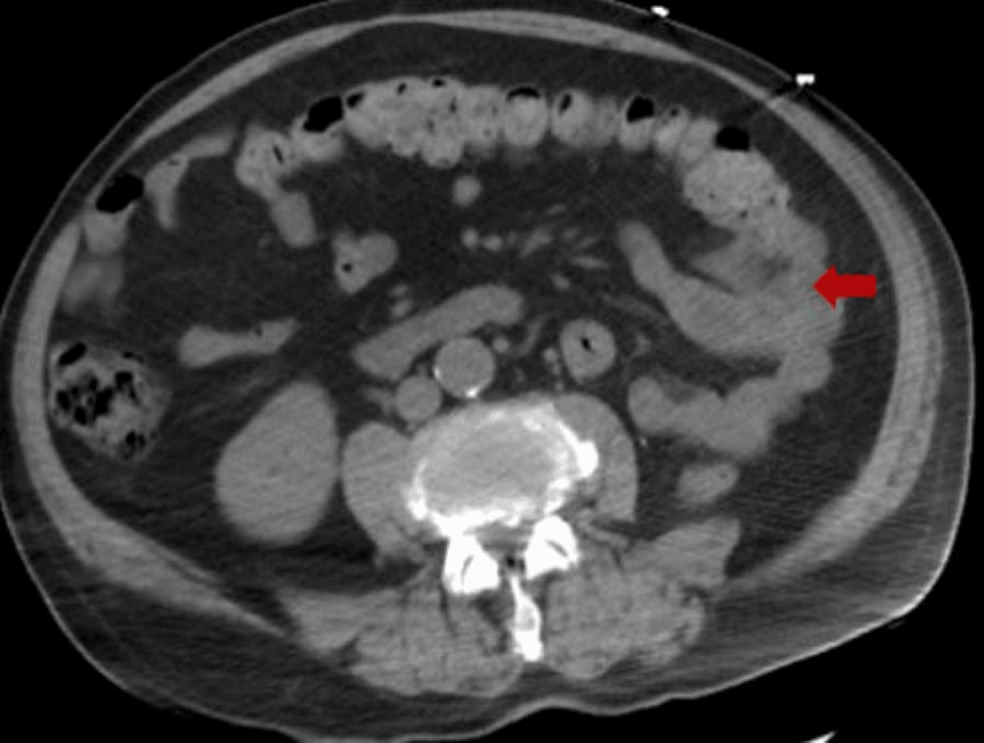
CT of the abdomen demonstrating colonic mass at the junction of the splenic flexure and proximal descending colon. CT, computed tomography

We describe an 87-year-old male with a past medical history of essential hypertension, hyperlipidemia, RCC with left-sided nephroureterectomy, partial adrenalectomy, and perinephric lymph node dissection three years ago. He presented to the emergency department (ED) with melena for one week, associated with generalized abdominal pain. The pain was more pronounced in the left upper quadrant, intermittently going on for a few weeks. He denied vomiting, hematochezia, diarrhea, or constipation. He mentioned having a colonoscopy six years ago, which was unremarkable. He denied recent use of nonsteroidal anti-inflammatory drugs or anticoagulants and reported no family history of colon cancer. His laboratory investigations revealed a white blood cell (WBC) count of 7400/mm^3^, hemoglobin of 9.6 g/dL, platelet count of 261000/uL, sodium of 143 mmol/L, potassium of 4 mmol/L, chloride of 110 mmol/L, bicarbonate of 24 mmol/L, blood urea nitrogen of 30 mg/dL, creatinine of 1.2 mg/dL, total bilirubin of 0.6 mg/dL, aspartate aminotransferase (AST) of 29 units/L, alanine aminotransferase (ALT) of 20 units/L, carcinoembryonic antigen (CEA) of 1.1 ng/mL, and cancer antigen 19-9 of 17.9 units/mL. Stool occult blood was positive. Computed tomography (CT) of the abdomen was significant for new lung nodules in the lower lobes measuring up to 1.2 cm, stable postsurgical changes related to prior left nephrectomy, and colonic mass at the junction of the splenic flexure and proximal descending colon (Figure 1).

**Figure 2:**
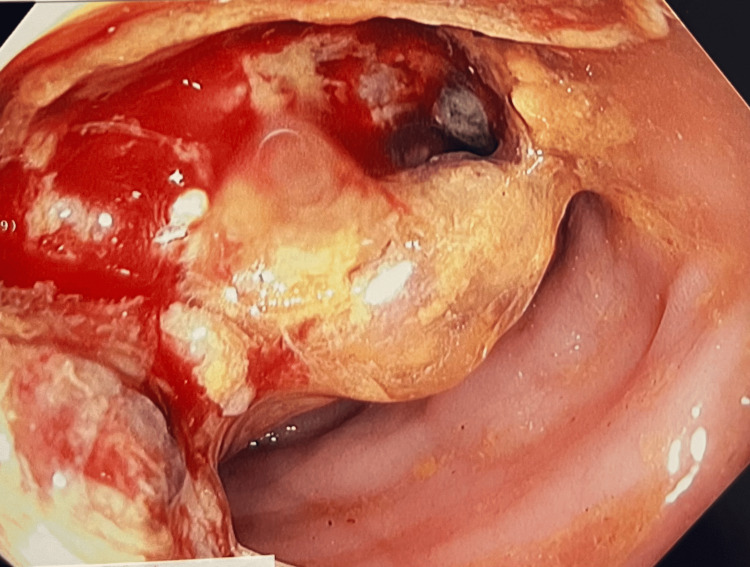
Colonoscopy showing necrotic appearing and friable mass in the proximal descending colon.

A colonoscopy was performed, which revealed mild diverticular disease, hemorrhoids, and a necrotic appearing friable mass in the proximal descending colon (Figure 2).

**Figure 3:**
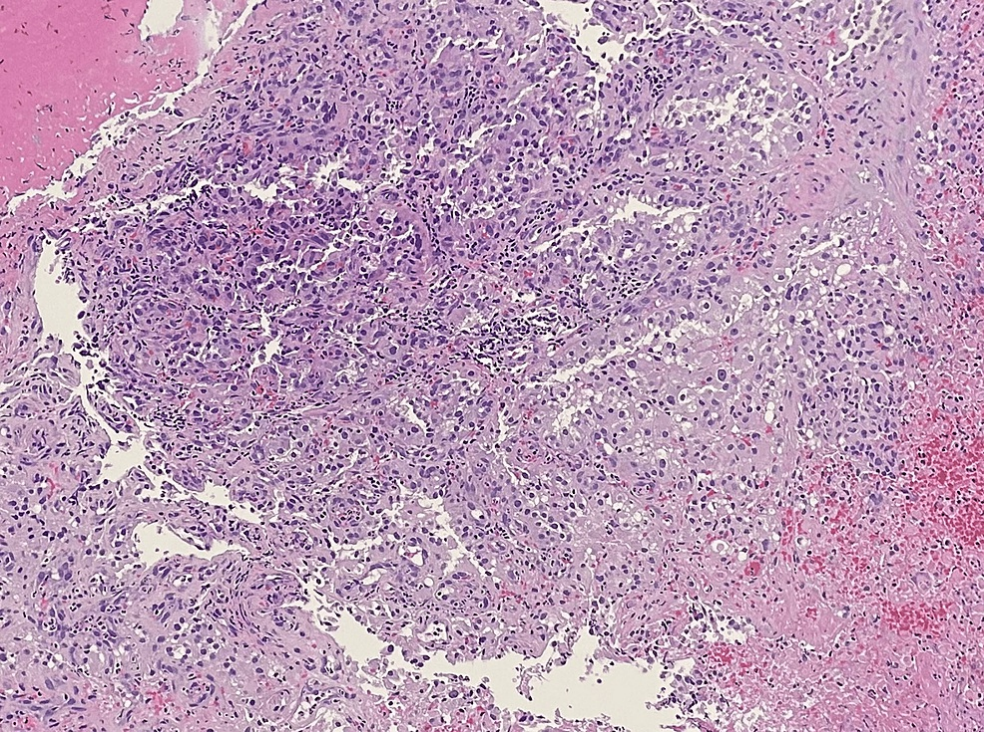
Histopathology of colonic mass demonstrating tumor cells with clear cytoplasm and atypical nuclei.

The pathology of the mass revealed proliferated atypical cells positive for paired box 8 (PAX8), cluster of differentiation 10 (CD10), RCC, and pan-cytokeratin and negative for caudal-type homeobox 2 (CDX2), thyroid transcription factor-1 (TTF-1), and cluster of differentiation 68 (CD68), consistent with metastatic RCC (Figure 3).

Therefore, the patient was referred to colorectal surgery and oncology for further evaluation.

## Discussion

RCC, a primary kidney cancer, predominantly occurs in adulthood with a higher prevalence in men, typically in the sixth and seventh decades of life [7]. While the majority of these tumors are sporadic, 4% of them have a hereditary component with association with specific syndromes like tuberous sclerosis, hereditary leiomyoma, Von Hippel-Lindau disease, and renal oncocytoma [1,8]. Given the lethality of RCC, survival is mainly contingent on the stage at the time of diagnosis. For instance, patients diagnosed with stage I disease exhibit a five-year survival rate of approximately 93%, whereas it is only 12% in patients diagnosed with stage IV [9]. The patient described in this case was diagnosed with stage III cancer before curative surgery.

The recurrence rate is 20-40% in RCC patients following surgical resection of primary tumor [1]. While most of these recurrences tend to occur within the initial five years post-curative surgery, a small percentage of patients experience late recurrences beyond this timeframe [10]. In this patient, recurrence happened three years after the surgical excision of the primary tumor.

Metastatic dissemination in RCC can occur through hematogenous, lymphatic, or direct invasion, and the likelihood of metastatic spread rises with tumor size. Even in the early stage of cancer, distant and lymph node spread can be observed, with metastatic risk escalating with the size of the tumor [11]. Due to this increased risk of metastasis, both the American Urology Association (AUA) and the National Comprehensive Cancer Network (NCCN) recommend regular surveillance for an initial five years after surgical resection of the tumor [1]. The treatment of choice for both oligo-metastatic disease and solitary metastatic disease is surgical intervention [12]. Based on this, the patient had undergone a left-sided nephroureterectomy, partial adrenalectomy, and perinephric lymph node dissection on initial diagnosis.

The most prevalent cancers that metastasize to the colon include stomach cancer, malignant melanoma, and breast cancer. Based on the literature, it is unusual for renal cell cancer to spread to the gut, but when it does metastasize, it can affect any part of the gastrointestinal tract. However, colonic metastasis is less common than metastasis to small bowel and stomach [3]. There is no distinct hematogenous or lymphatic route that could adequately explain the spread of RCC to the colon. The location of metastasis within the colon also differs, with rectosigmoid, splenic flexure, and transverse colon being the most commonly affected areas [1]. Our patient was found to have metastasis to the proximal descending colon, which is an unusual location for metastasis within the colon.

## Conclusions

We describe an unusual case of metastatic RCC to the descending colon in a patient who presented with generalized abdominal pain and melena. The patient underwent curative surgery for RCC three years ago. This case highlights the fact that colonic tumors can arise from metastatic RCC, especially in patients with a history of renal cancer or nephrectomy. Therefore, such patients, when present with gastrointestinal bleeding or abdominal pain, should undergo detailed screening through imaging and physical examination. The case also shows that the recurrence pattern of RCC is unpredictable, and such patients should be educated on long-term follow-up even after surgical removal of the primary tumor.
